# Training-induced behavioral and brain plasticity in inhibitory control

**DOI:** 10.3389/fnhum.2013.00427

**Published:** 2013-08-01

**Authors:** Lucas Spierer, Camille F. Chavan, Aurelie L. Manuel

**Affiliations:** ^1^Neurology Unit, Department of Medicine, Faculty of Sciences, University of FribourgFribourg, Switzerland; ^2^Psychiatry Unit, Department of Medicine, Faculty of Sciences, University of FribourgFribourg, Switzerland; ^3^Laboratory of Cognitive Neurorehabilitation, Department of Clinical Neurosciences, Medical School, University of GenevaGeneva, Switzerland

**Keywords:** plasticity, inhibitory control, training, rehabilitation, frontal

## Abstract

Deficits in inhibitory control, the ability to suppress ongoing or planned motor or cognitive processes, contribute to many psychiatric and neurological disorders. The rehabilitation of inhibition-related disorders may therefore benefit from neuroplasticity-based training protocols aiming at normalizing inhibitory control proficiency and the underlying brain networks. Current literature on training-induced behavioral and brain plasticity in inhibitory control suggests that improvements may follow either from the development of automatic forms of inhibition or from the strengthening of top-down, controlled inhibition. Automatic inhibition develops in conditions of consistent and repeated associations between inhibition-triggering stimuli and stopping goals. Once established, the stop signals directly elicit inhibition, thereby bypassing slow, top-down executive control and accelerating stopping processes. In contrast, training regimens involving varying stimulus-response associations or frequent inhibition failures prevent the development of automatic inhibition and thus strengthen top-down inhibitory processes rather than bottom-up ones. We discuss these findings in terms of developing optimal inhibitory control training regimens for rehabilitation purposes.

## Inhibitory control

### Measures of inhibitory control

Inhibitory control refers to the ability to suppress ongoing or planned motor or cognitive processes and enables adapting to rapidly changing situations (Aron et al., 2004; Aron, 2007). While in the current review, we focus on the inhibitory control of motor responses, it is worth noting that a large variety of experimental paradigms involving—at least partly—inhibition processes have been used in training studies [e.g., Flanker, Stroop, or antisaccade tasks; reviewed in Aron (2011)]. Because these tasks involve many different components in addition to inhibitory control [attention, working memory (WM), etc], they have been presented as concerning the more general “executive control” construct. Further work on training-induced plasticity of inhibition focused on training self-control (e.g., Muraven, 2010). The stop-signal task (SST; Lappin and Eriksen, 1966) and the Go/NoGo task (Donders, 1969) involve very directly motor inhibitory control and were thus primarily used to train this aspect.

The SST consists of a speeded discrimination task in which responses to the stimuli have to be canceled when a stop signal is presented (Logan et al., 1984; Band et al., 2003). The shorter the delay between the stimulus and the stop signal, the higher is the probability of successfully inhibiting an ongoing response. SST performance is usually indexed by stop-signal reaction time (SSRT) calculated as the delay from the stop signal at which the stopping process successfully suppresses the motor response to the preceding go signal (e.g., Logan, 1994). By contrast, in the Go/NoGo task, participants have to respond as fast as possible to a set of stimuli, while withholding their responses to another set of stimuli. The number of responses to NoGo stimuli (false alarms) and reaction times to Go stimuli index inhibition performance.

### Neural bases of inhibitory control

Converging neuroimaging and clinical data indicate that inhibitory control depends on a cortico-subcortical network comprising the inferior frontal gyrus (IFG), the pre-supplementary motor area (preSMA), and the basal ganglia (for reviews see, e.g., Aron, 2007; Chambers et al., 2009; Chikazoe, 2010). The inhibitory processes typically take place around 100–200 ms after the onset of the stop-stimulus (Falkenstein et al., 1995, 1999; Rubia et al., 2001; Aron et al., 2004). Current models of inhibitory control suggest that action elicitation (“Go”) and cancelation (“Stop”) processes are mostly independent up to their final stage, where they interact to ultimately suppress prepotent or actual behavioral responses if a stop signal was presented (Hanes et al., 1998; Aron et al., 2007; Boucher et al., 2007). Once processed by early sensory cortices, the information about the Go signals is relayed to the premotor area, which activates primary motor areas via the striatum, pallidum, and thalamus (Aron and Poldrack, 2006). The information about Stop signals is relayed to the inferior frontal cortices and/or the pre-SMA; these structures generate the inhibition via the basal ganglia input nuclei [notably the subthalamic nucleus (STN)], which connects to the globus pallidus mediating response inhibition by suppressing the final stages of the Go process (Aron et al., 2007; Chambers et al., 2009; Aron, 2011).

### Domain-general inhibitory control mechanism

Latent variables analyses suggest that in addition to task-specific processes, there is a common, domain-general inhibitory control mechanism involved across all inhibitory tasks (Brocki and Bohlin, 2004; Friedman and Miyake, 2004), and contributing to many higher-order cognitive abilities including, for example, abstract reasoning, the resolution of complex problem, and decision making (Chiappe et al., 2000; Houde et al., 2000; Handley et al., 2004; Viskontas et al., 2004; Cain, 2006; De Neys and Everaerts, 2008). The existence of a domain-general inhibitory control mechanism is further supported by functional neuroimaging evidence for the involvement of the same fronto-striatal network in the inhibition of various cognitive and motor processes. The pre-SMA, IFG, and STN have indeed been shown to support the inhibition of eye movements (Chikazoe et al., 2007), linguistic processes (Xue et al., 2006, 2008), thoughts, memories and even emotions (Jonides et al., 1998; Depue et al., 2007; Dillon and Pizzagalli, 2007; Mitchell et al., 2007). Of note, however, whether this network is solely involved in inhibitory control or more general attentional phenomena remains debated (e.g., Hampshire et al., 2010).

## Plasticity of inhibitory control

With regard to the considerable research efforts having addressed the neural underpinnings of inhibitory control, whether this function can be improved with training and whether the underlying brain networks are subject to plastic changes remain surprisingly unknown.

Broadly defined, “plasticity” refers to experience-dependent modifications of the behavior and of its underlying anatomo-functional brain organization. These modifications support the acquisition of new skills, the improvement of already acquired abilities and the recovery of functional deficits or disorders (e.g., Kelly and Garavan, 2005). Training-induced behavioral and brain plastic changes have been demonstrated at the level of various executive functions. For instance, training WM or planning abilities improves performance (Dahlin et al., 2008; Jaeggi et al., 2008; Li et al., 2008; Von Bastian et al., 2013) and modifies the activity of fronto-parietal or medial frontal brain network, respectively (Beauchamp et al., 2003; Olesen et al., 2004; Dahlin et al., 2008; Klingberg, 2010).

**Figure 1:**
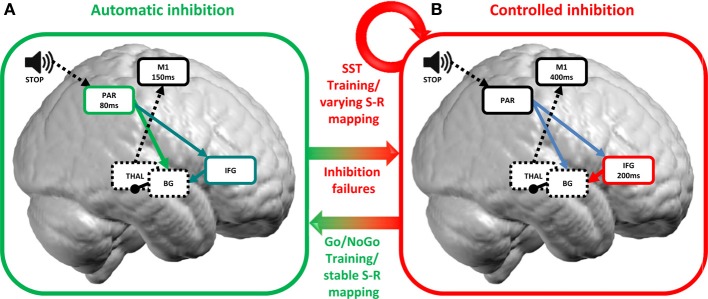
**Two mechanisms of training-induced plasticity of inhibitory control**. Inhibition stimuli are conveyed to sensory areas processing stimuli features at ca. 80 ms within parietal brain regions (Hyde et al., 2008; Spierer et al., 2008). **(A)** In conditions of stable S-R mapping, as for the Go/NoGo task, participants switch from a controlled to an automatic inhibition mode with training. Automatic inhibition develops in parietal areas at ca. 80 ms and shortcuts top-down inputs from the IFG (green arrow; although see Lenartowicz et al., 2011 for evidence of a role for the IFG in automatic inhibition; blue arrows) in turn leading to faster inhibition (ca. 150 ms; calculated as the mean RT −100 ms which corresponds to the latency of M1 initiation before motor execution; Thorpe and Fabre-Thorpe, 2001). **(B)** When S-R mapping varies (as in e.g., SST), top-down, controlled inhibition is modulated by training around 200 ms in the IFG. The IFG then activates subcortical basal ganglia (red arrow) which in turn inhibits the thalamocortical output and suppresses motor execution in M1. Error commission allows shifting from fast automatic to slow top-down controlled forms of inhibition. PAR, parietal; M1, primary motor cortex; IFG, inferior frontal gyrus; BG, basal ganglia; THAL, thalamus; S-R mapping, stimulus-response mapping. Arrows indicate excitatory connections and rounds inhibitory connections. Full lines indicate cortical structures and dashed lines indicate subcortical structures.

While the plasticity of WM training has received much attention within the field of executive functions, only few studies investigated plasticity of inhibitory control (see Table 1). Hypotheses about the neuroplastic changes underlying inhibitory control, therefore, are only beginning to emerge. The literature so far documents two main mechanisms of training-induced behavioral and brain plasticity of inhibitory control: the development of bottom-up, automatic forms of inhibition and the optimization of top-down, controlled forms of inhibition (Figure 1).

**Table 1:** **Studies involving inhibitory control training**.

**Study**	**Training task**	**Method**	**Total # trials in the training**	**Estimated training duration (in minutes)**	**Effect of the training on the trained task**	**Transfer of the effects of training to untrained conditions**
Manuel et al. (2013)	SST	EEG	1020	60	SSRT ↓	nt
					Fronto-striatal ↓	
Manuel et al. (2010)	GNG	EEG	528	40	Go RT ↓, FA ↑	nt
					Parietal ↓	
Benikos et al. (2013)	GNG	EEG	830	40	Go RT ↓, FA ↑ or ↓	nt
					P3 ↑, N2 ↓	
Bowley et al. (2013)	GNG	EEG	80	4	nr	To alcohol intake
Jodo and Inoue (1990)	GNG	EEG	1200	150 (25 min per day for 6 days)	Go RT ↓	nt
					P3 Latency ↓	
Schapkin et al. (2007)	GNG	EEG	6000	240 (16 min per day for 15 days over 3 weeks)	FA ↓	nt
					N2 ↑	
Houben et al. (2012)	GNG	BHV	320	20	nr	To alcohol intake
	SST		256	30		To implicit attitudes
Houben (2011)	SST	BHV	288	10–15	nr	To eating
Houben and Jansen (2011)	GNG	BHV	320	15	nr	To eating
Houben et al. (2011)	GNG	BHV	80	4	nr	To alcohol intake
Verbruggen et al. (2012)	SST	BHV	720	30	↓ Monetary risk	To gambling
Thorell et al. (2009)	GNG	BHV	nr	375 (15 min per day for 25 days)	*↑ performance over time in GNG (↓ commission errors)	None
	SST				
	Flanker				
					*↑ performance over time in Flanker's task
					* No improvement over time in SST
Veling et al. (2011)	SST	BHV	120	6	*↑ RT after presentation of palatable food	To caloric food consumption
			72	3.5	nr	
Logan and Burkell (1986)	SST	BHV	4320	360	SSRT ↓	nt
Guerrieri et al. (2008)	SST	BHV	192	20	* SSRT ↓ for normal weighted	No transfer to food intake
					* SSRT ↑ for overweighed	
Guerrieri et al. (2012)	SST	BHV	600	nr	nr	No transfer to food intake in the “inhibition” condition
Cohen and Poldrack (2008)	SST	BHV	7200	180 (3 times 60 within a week)	no effect on SSRT	nt
Lenartowicz et al. (2011)	SST	BHV	600	25	↑ Pars triangularis of rIFG to go trials associated with inhibition	nt
		fMRI				
Chiu et al. (2012)	GNG	TMS	Learning: 864	Learning: 30	* ↓ MEPs for stimuli associated with stopping	nt
		BHV	TMS: 288	TMS: 20	
					*↑ Motor suppression in inconsistent condition for subjects who learned the most during training
			TMS: 720	50	↓ MEPs for NoGo trials in the midphase of learning	
Johnstone et al. (2012)	GNG	BHV	4500	450 (15–20 per day for 25 days)	↑ GNG performance (children reached higher difficulty level)	ADHD children
		EEG			
						* To ADHD symptoms
						* To oddball
						* To flankers
						* EEG: To beta activity
						Healthy children
						* Not to oddball
						* To flankers
						* EEG: To beta activity

nt, not tested; nr, not reported or not possible to estimate; SST, stop-signal task; GNG, Go/NoGo task; BHV, behavioral; EEG, electroencephalography; MEP, motor evoked potential; IFG, inferior frontal gyrus; FA, false alarms; RT, response time; SSRT, stop-signal reaction time; ADHD, attention deficit hyperactivity disorder.

### The development of automatic, bottom-up forms of inhibition

In a series of psychophysical experiments on inhibitory control training, Verbruggen and Logan (2008) showed that if Go and NoGo stimuli were respectively consistently associated with Go and NoGo goals during inhibitory control training with a classical Go/NoGo task, automatic (i.e., unintentional) forms of inhibition develop with practice. Based on evidence that when Go/NoGo stimulus-response mapping rules are reversed after a practice phase, response to Go stimuli are slowed, the authors advance that with training, automatic processes progressively replaced top-down controlled processes to inhibit prepotent motor responses. After the training, NoGo goals were automatically activated by NoGo stimuli and thus higher-order, top-down forms of control were no longer required to resolve the Go/NoGo task. In turn, inhibition was faster and Go/NoGo performance improved overall (see also Shiffrin and Schneider, 1977).

Recent work from our group provided insights into the neural mechanisms underlying the development of automatic forms of inhibitory control. Using a training paradigm involving stable stimulus-response mapping rules, we contrasted event-related evoked potentials (ERPs) to “Go” and “NoGo” stimuli between the beginning vs. the end of a 40 min auditory spatial Go/NoGo training and during passive listening of the same stimuli before vs. after the training session (Manuel et al., 2010). Consistent with the behavioral studies by Verbruggen and Logan (2008), we showed that training improved Go/NoGo performance. During the active training session, electrophysiological responses to NoGo, but not Go, stimuli engaged distinct brain networks at the beginning vs. the end of the training ca. 80 ms after stimulus onset, driven by a decrease in the activity of left parietal cortices. This modulation in brain activity correlated positively with the behavioral improvement in inhibitory control. As for the processing of the Go and NoGo stimuli in passive listening conditions, the training modulated ERPs in response to Go stimuli at ca. 50 ms, due to decreased right anterior temporo-parietal activity. These results support the automaticity hypothesis by Logan and colleagues: Driven by repeated and stable associations between NoGo stimuli and response withholding, inhibitory control training resulted in a progressive disengagement of frontal top-down inputs for resolving the inhibitory task in favor of fast automatic forms of inhibition (Shiffrin and Schneider, 1977; Logan, 1988; Verbruggen and Logan, 2008). Supporting this hypothesis, Manuel et al. (2010) reported that automatic inhibition was implemented within parietal areas, a region interfacing sensory representations of the feature discriminating auditory spatial Go and NoGo stimuli and response-related motor commands (Deiber et al., 1991, 1996; Decety et al., 1992; Andersen et al., 1997). Interestingly, Lenartowicz et al. (2011) reported a slowing down of response time for Go stimuli that were previously paired with a stop stimulus in a training phase. This automatically triggered inhibition was accompanied by an increase in IFG activity, suggesting that automatic inhibition may still be mediated by higher-order frontal areas. However, Manuel et al. (2010) used a Go/NoGo task and Lenartowicz et al. (2011) an SST; the inhibitory mechanisms in the two tasks might partly differ because in SST, ongoing motor responses have to be inhibited while in Go/NoGo, participants have to suppress prepotent responses.

Collectively, these studies suggest that behavioral improvements in inhibitory control with training regimens involving unvarying stimulus-response mappings are supported by the development of fast, automatic forms of response inhibition instead of relying solely on the reinforcement of top-down inputs from frontal areas (Manuel et al., 2010; Figure 1). Of note, the automatization of inhibitory control with practice is at odds with traditional views of inhibitory control as an inherently top-down executive process (Aron et al., 2004). Perhaps counter-intuitively, inhibitory control can be automatic and driven by brain areas usually thought to trigger movements. These results further suggest that top-down executive frontal areas are not always necessarily required to control behavior, or at least that the engagement of these inhibitory areas can be driven automatically by specific stimuli (Lenartowicz et al., 2011).

### Modification of controlled, top-down forms of inhibition

Based on our findings in Manuel et al. (2010), we hypothesized that when inhibitory control training is based on a task involving inconsistent mappings between stimulus and response, automatic processing would not develop. Rather, only top-down control mechanisms would be constantly solicited during the training phase, and thus ultimately modified by practice (Shiffrin and Schneider, 1977; Verbruggen and Logan, 2008). Such a pattern would, for instance, be induced by training with an SST because in SST, Go goals are engaged in all trials and must only sometimes be subsequently canceled.

To test this hypothesis, we contrasted event-related potentials to Go stimuli at the beginning vs. the end of a 1-h auditory stop signal training session (Manuel et al., 2013). The training improved inhibitory performance as evidenced by a significant decrease in stop-signal reaction time, i.e., the latency of the stop process. At the neurophysiological level, we observed plastic modification of a right lateralized fronto-basal network comprising the IFG, the pre-SMA and the basal ganglia at ca. 200 ms post-stimulus onset, suggesting that top-down executive networks were modified with SST training (Figure 1). Alternatively, such modification could also reflect a change in proactive control strategy since we contrasted responses to Go stimuli which were not consistently paired with going or stopping.

In addition to inconsistent stimulus-response associations, a second parameter may be manipulated to promote the modification of top-down mechanisms over the development of automatic inhibition: the difficulty of the task. An increase in the difficulty to inhibit prepotent or ongoing responses indeed increases inhibition failures (commission errors), which in turn lead to the (re)engagement of controlled forms of inhibition. During inhibitory tasks, post-error shifts to more cautious response modes translate into switches from fast automatic to slow, top-down controlled forms of inhibition (Manuel et al., 2012), which could account for post-error slowing effects (Rabbitt, 1966; Notebaert et al., 2009). Controlled forms of inhibition would be prominently involved in tasks generating many errors and thus training with difficult task would likely modify top-down mechanisms. Consistent with this finding, Benikos et al. (2013) showed that while training on a moderately difficult Go/NoGo task improved inhibitory control performance, training with lower or higher inhibitory demands failed to induce, respectively, an improvement or a decrease in performance. Moreover, Benikos et al. (2013) reported that training-induced improvements in the moderate inhibition load condition followed from an increase in the P3 evoked potential, compatible with a modification of top-down inhibition mechanisms.

### Neurophysiological mechanisms of training-induced functional changes

While current literature clearly suggests that inhibitory control is subject to fast plastic changes, the underlying neurophysiological mechanisms remain largely unresolved. In the studies reported above, performance improvements were accompanied by a decrease in the activation strength of inhibition-related stimuli (Manuel et al., 2010, 2013). Although very speculative, such pattern of training-induced functional changes might follow from an increase in synaptic efficacy and/or in neural efficiency (Haier et al., 1992), yielding to a decrease of the number of neurons responding during the trained task, which would in turn increase the speed of inhibition processes (Schoups et al., 1998; Poldrack, 2000; Song et al., 2002; Kelly and Garavan, 2005). The refinement of inhibitory control network could also follow from strengthening in the synaptic connections between the neural ensembles involved in the inhibition and in a decrease in the connections with less critical regions (Galvan, 2010). Further studies should also determine whether and how inhibitory control training may induce structural modifications of the involved brain areas. Modification of gray matter volume or density (e.g., following from neuro- or synapto-genesis), as well as changes in neuronal morphology or in glial cells have indeed been demonstrated following the training of many cognitive and motor skills (Zatorre et al., 2012; Thomas and Baker, 2013 for reviews). Similarly, training-induced plasticity has been shown at the level of white matter microstructure (Zatorre et al., 2012).

### Generalization of the effects of training

We have reported above how specific inhibitory control training parameters promote either the development of automatic forms of inhibition or the modification of top-down controlled inhibition. Critically, each of the mechanisms of training-induced plasticity also determines the extent to which the effects of the training will generalize to other stimuli, conditions, or tasks. One could indeed hypothesize that if top-down control mechanisms are modified by training, the effects of practice would transfer to other untrained conditions or tasks supported by the same fronto-basal brain network. In contrast, because automatic inhibition consists in associations between specific stimuli and inhibition goals, the effects of training regimen promoting the development of automatic forms inhibition would unlikely generalize to untrained stimuli. Training-induced modifications of automatic inhibition processes might be, however, advantageous in the remediation of traits characterized by highly automatized (inappropriate) responses as in, e.g., impulsivity (Marteau et al., 2012).

Empirical support for these assumptions comes from Thorell et al. (2009), who showed that training on a Go/NoGo with consistent S-R mapping rules, improved inhibitory control on the trained Go/NoGo task, but did not transfer to other inhibitory control tasks (SST or Flanker), nor to other executive tasks including, e.g., WM or problem solving tasks. The fact that Go/NoGo training improved performance without transferring to other executive processes indicates that automatic, forms of inhibition, but not top-down controlled inhibition developed. In contrast, SST training led to larger transfer of the effect of training supporting that it modified higher-order top-down processes. Another example of distant transfer of the effects of inhibitory control training with stop signal task comes from Verbruggen et al. (2012), who showed that a brief training on an SST diminished risky behavior during a subsequent monetary gambling task. This study indicates that training executive processes at the motor level (stop signal task) transfer to other, non-motor, decision making tasks. Specifically designed training studies, examining systematically the generalization patterns of training regimens promoting either the development of automatic or controlled inhibition are required to elucidate the question of the transfer of the effects of inhibitory control training.

Several other studies demonstrate that when the domain-general inhibitory control network is modified by training, the effects of the training will influence subsequent complex behavior involving the same inhibitory control component (Houben and Jansen, 2011; Houben et al., 2011, 2012; Veling et al., 2011; Jones and Field, 2013). Houben et al. (2011) trained alcohol drinking participants on a Go/NoGo task. In one group, Go stimuli were consistently paired with alcohol-related stimuli, whereas in the second group, the NoGo stimuli were paired with alcohol-related stimuli. The results revealed that participants in the second group had increased negative attitudes toward alcohol and significantly reduced weekly alcohol consumption. The effects of inhibitory control training have also been tested on food consumption. Houben (2011) trained healthy participant with an SST in which they had to respond as fast as possible to Go stimuli and to withhold their response when a stop signal was presented after a Go signal. In an “inhibition” condition aiming at improving the inhibition of responses to food, high-calorie food items were systematically paired with a stop signal. In an “impulsivity” condition aiming at strengthening impulses to food, the high-calorie food item was never paired with a stop signal. The results revealed that immediately after the training, participants in the “inhibition” condition consumed significantly less high-calorie food than the participants who underwent the “impulsivity” condition. Similar effects were found by Houben and Jansen (2011), who showed that using “chocolate” items as NoGo stimuli decreased subsequent chocolate consumption as compared to conditions where chocolate items were presented as Go stimuli. Finally, Veling et al. (2011) showed that food-related SST training also helped chronic dieters to control behavior to food.

The reciprocal effect has also been confirmed by studies showing that practice on complex tasks involving an inhibitory control component improves performance on basic inhibitory control tasks. For instance, Di Russo et al. (2006) showed that fencing athletes were more proficient than control populations in Go/NoGo tasks.

### Rehabilitation of inhibition-related disorders

Evidence for training-induced improvement in inhibitory control and for the modification of the underlying fronto-basal network suggest that practicing inhibition tasks might help recovering for inhibition-related pathologies (e.g., Johnstone et al., 2012). Inhibitory control deficits and abnormal anatomic variations at various levels of the fronto-basal network supporting inhibitory control have indeed been advanced as constituting a causal factor or at least as being strongly associated with the emergence of psychiatric disorders including, e.g., addiction, schizophrenia, compulsive disorders, obesity, or ADHD (Badcock et al., 2002; Aron and Poldrack, 2005; Holroyd et al., 2005; Garavan and Hester, 2007; Ruchsow et al., 2007, 2008; Eisenberg and Berman, 2010; McLoughlin et al., 2010) as well as related pathological traits such as impulsivity (Aron and Poldrack, 2005; Knoch et al., 2006; Chambers et al., 2009).

## Future perspectives

Many questions remain open within the field of inhibitory control plasticity. For instance, the long-term effects of inhibitory control training, and the effects of long training sessions, remain unresolved. Houben et al. (2011) showed evidence for an effect of short Go/NoGo training on drinking behavior up to 1 week following training, suggesting that the improvements may persist over time. Regarding the length of training sessions, there is no direct evidence for a greater improvement with long rather than short training regimen, and the interaction between training duration and the persistence of the effects of training remain to be explored.

We have proposed a few task parameters favoring the reinforcement of controlled rather than automatic inhibitory processes, which in turn would promote the generalization of the effect of inhibitory control training to other conditions and tasks. These proposals remain, however, mostly speculative and need further investigations. In this regard, the relevance of inhibitory control training for the rehabilitation of inhibition-related disorders also needs to be further established.

Collectively, current literature on inhibitory control plasticity suggests that training inhibitory control may constitute a promising tool for the rehabilitation of many inhibition-related psychiatric and neurological disorders.

### Conflict of interest statement

The authors declare that the research was conducted in the absence of any commercial or financial relationships that could be construed as a potential conflict of interest.
